# Increased seroprevalence of lymphocytic choriomeningitis virus infection in mice sampled in illegal waste sites

**DOI:** 10.1186/1756-3305-7-S1-O31

**Published:** 2014-04-01

**Authors:** D Duh, E Varljen-Buzan, S Hasic, R Charrel

**Affiliations:** 1 Science and Research Centre, University of Primorska, Koper - Capodistria, Slovenia; 2 Faculty of Mathematics, Natural Sciences and Information Technologies University of Primorska, Koper - Capodistria, Slovenia; 3 Faculty of Medicine, Aix-Marseille University, Marseille, France

## 

Lymphocytic choriomeningitis virus (LCMV), the prototypic member of the Arenaviridae family, was isolated from a fatal case of aseptic meningitis during the St. Louis encephalitis epidemic in 1933. Although it was among the first isolated human pathogenic viruses, LCMV remained less attractive through years due to its lack of clinical importance even though it is an important teratogen causing a severe and permanent brain injury in newborns. Recently, this old neglected virus found a new niche in immunosuppressed solid organs recipients. The natural host and reservoir of LCMV is a common house mouse, *Mus musculus*. Zoonotic transmission of LCMV to humans occurs worldwide by direct route with mice secretions. LCMV has been also isolated from several arthropods including fleas, *Culicoides *flies, *Aedes *mosquitoes and ticks, but it is believed that arthropods play a minor role in LCMV transmission. However, in habitats where mice and arthropod vectors are abundant, a vector transmission of LCMV could be important. The illegal waste sites present optimal conditions for spread of rodents, common house mice and rats in particular. Therefore, we tested 83 samples of *Mus *or *Apodemus *species sampled on illegal waste sites in Slovenia and Croatia. LCMV infection was determined with IFA and RT-PCR techniques. IFA slides and positive controls were kindly provided by Remi Charrel. The presence of IgG against LCMV was detected in 47% and 16% sera samples of *Mus musculus *and *Apodemus *species, respectively. Total RNA was extracted from spleen using RTP Pathogen Kit (Invitek-Stratec) and tested with nested RT-PCR specific for amplification of LCMV genome. Amplicons are in process of sequencing to obtain the nucleotide sequence and confirm the presence of LCMV.

Since the seroprevalence studies in Europe revealed up to 17% prevalence of LCMV infection in mice and voles sampled in natural host habitat, we concluded that illegal waste sites accelerate the spread of rodent-borne pathogenic LCMV.

